# Genome-wide identification and characterization of pectin methylesterase inhibitor gene family members related to abiotic stresses in watermelon

**DOI:** 10.3389/fpls.2024.1454046

**Published:** 2024-09-17

**Authors:** Siyu Zhang, Xinhao Yuan, Jiahao Duan, Jun Hu, Chunhua Wei, Yong Zhang, Jiafa Wang, Chao Li, Shengcan Hou, Xiaodan Luo, Junhua Li, Xian Zhang, Zhongyuan Wang

**Affiliations:** ^1^ State Key Laboratory of Crop Stress Biology in Arid Areas, College of Horticulture, Northwest A & F University, Xianyang, China; ^2^ Research Institute of Grape and Melon of Xinjiang Uyghur Autonomous Region, Turpan, China; ^3^ Kaifeng Academy of Agriculture and Forestry Sciences, Kaifeng, China

**Keywords:** watermelon, PMEI family, whole genome identification, abiotic stresses, expression analysis

## Abstract

Pectin is a vital component of plant cell walls and its methylation process is regulated by pectin methylesterase inhibitors (PMEIs). PMEIs regulate the structural and functional modifications of cell walls in plants and play an important role in plant processes such as seed germination, fruit ripening, and stress response. Although the PMEI gene family has been well characterized in model plants, the understanding of its molecular evolution and biological functions in watermelon remains limited. In this study, 60 ClPMEI genes were identified and characterized, revealing their dispersion on multiple chromosomes. Based on a systematic developmental analysis, these genes were classified into three subfamilies, which was further supported by the exon, intron, and conserved motif distribution. Analysis of cis-elements and expression patterns indicated that ClPMEIs might be involved in regulating the tolerance of watermelon to various abiotic stresses. Moreover, distinct ClPMEI genes exhibit specific functions under different abiotic stresses. For example, *ClPMEI51* and *ClPMEI54* showed a significant upregulation in expression levels during the late stage of drought treatments, whereas *ClPMEI3* and *ClPMEI12* displayed a significant downregulation under low-temperature induction. Subcellular localization prediction and analysis revealed that the ClPMEI family member proteins were localized to the cell membrane. This study provided an important foundation for the further exploration of the functions of ClPMEI genes in watermelon.

## 1 Introduction

The cell wall is a complex dynamic network structure that performs numerous essential functions, including the regulation of plant growth and development, intercellular communication, stress response, and immune resistance (Keegstra, 2010). Pectin, a major component of the plant cell wall, is a key determinant of plant cell morphology (Carpita and Gibeaut, 1993). Pectin is a complex polysaccharide polymer with a skeleton composed of galacturonic acid (GalA) residues (Ridley et al., 2001). Based on the structure of their backbones and the diversity of their side chains, pectin can be classified into five distinct subclasses: xylogalacturonan, homogalacturonan (HG), rhamnogalacturonan I (RG-I), rhamnogalacturonan II (RG-II), and apiogalacturonan (Wolf et al., 2009). HG is the most abundant pectic polysaccharide in primary cell walls, and the degree of methylesterification (DM) determines the biomechanical properties of the cell wall (Pérez et al., 2000). This DM is controlled by the activity of endogenous proteins called pectin methylesterases (PMEs) (Juge, 2006; Pelloux et al., 2007). PMEs are involved in many physiological processes (Wu et al., 2018). PMEs also play a critical role in plant immunity (Del Corpo et al., 2020; Coculo et al., 2023; Corpo et al., 2024). In addition to its transcription level, protein modification, and endogenous pH, PME activity is also regulated by a class of multigene family coding pectin methylesterase inhibitors (PMEIs) (Wormit and Usadel, 2018).

Since the first report of a specific powerful glycoprotein inhibitor of PME in kiwi fruit, the PMEI gene family has been widely identified in several plant species (Coculo and Lionetti, 2022). To date, 79 PMEI genes have been identified in *Arabidopsis thaliana*, 49 in *Oryza sativa*, 95 in *Brassica oleracea*, and 42 in *Pyrus bretschneideri*, among others (Wang et al., 2013; Nguyen et al., 2016; Liu et al., 2018; Zhu et al., 2021). Furthermore, through the study of the structure of PMEI genes using crystallographic approaches, it was found that the three-dimensional structure of PMEI in *Arabidopsis* consists of four long α-helices arranged in an up-down-up-down topology, forming a four-helix bundle (Hothorn et al., 2004). The function of PMEI genes has been widely studied in several plants. PMEI genes have been reported to regulate the elongation of plant hypocotyls and stems by regulating pectin methylation and cell wall thickness. In *A. thaliana*, overexpression of *PMEI4* delayed the onset of hypocotyl growth, suggesting that *AtPMEI4* is involved in regulating hypocotyl growth (Pelletier et al., 2010). Similarly, overexpression of *OsPMEI28* resulted in a dwarfed phenotype of rice by inhibiting culm elongation and decreasing the cell wall thickness of culms (Nguyen et al., 2017). On the other hand, the pectin methylation regulated by *PMEI* genes is related to the process of pollen tube elongation, which depends on cell expansion (Jiang et al., 2005; Tian et al., 2006). The application of the purified proteins of *AtPMEI4* and *AtPMEI9* had distinct consequences on pollen tube elongation in *A. thaliana* (Hocq et al., 2017). Mature *A. thaliana* seeds possess mucilage composed of polysaccharides in coat epidermal cells, which is unnecessary for seeds in terms of germination (Western et al., 2000; Francoz et al., 2015). PMEI genes regulate the seed germination process by decomposing polysaccharides in seed epidermal mucilage (Müller et al., 2013). Significant direct evidence has been obtained to prove that PMEIs regulate the release of polysaccharides from seed coat epidermal cells through various studies. For instance, pmei 6 mutants in *A. thaliana* resulted in a delay in mucilage extrusion and an increase in PME activity in seeds (Saez-Aguayo et al., 2013). Additional evidence reported that *AtPMEI14* is also involved in regulating the decomposition of polysaccharide in seed epidermal mucous (Shi et al., 2018). The function of PMEI genes in pectin methylation was also reflected in the regulation of fruit ripening. In tomato, PMEIs modify fruit softening by regulating the spatial patterning of the distribution of esterified pectin in fruit (Reca et al., 2012).

Additionally, the PMEI gene family has the function of response to stress by modifying the plant cell wall structure. The plant cell wall is an important barrier against pathogen invasion (Hamann, 2015). Pathogens invade plants by secreting hydrolase to decompose pectin in the cell wall. PMEI genes regulate the infection degree of plant pathogens by regulating the level of pectin methylation in the cell wall (Lionetti, 2015; Raiola et al., 2011; Lionetti et al., 2017). After transferring a PMEI gene from kiwi fruit into wheat, it was found that wheat was less susceptible to pathogen invasion (Volpi et al., 2011). Similarly, plant cell wall architecture also plays an important role in responding to abiotic stresses such as water deficit, salt stress, and temperature extremes (Wormit and Usadel, 2018). Through transcription detection of the *CaPMEI1* gene in pepper, it was found that low temperature, drought stress, abscisic acid, and hydrogen peroxide induced its expression (An et al., 2008). However, overexpression of *CbPMEI1* and *PMEI13* in *A. thaliana* reduced cold under low-temperature stress (Chen et al., 2018). Under cold stress, leaf tensile stiffness, cell wall composition, and pectin content are crucial for freezing tolerance in plants (Solecka et al., 2008). Pectin in the cell wall under low temperatures helps reduce cell wall porosity, increases cell adhesion, and impedes ice propagation (Ashworth and Abeles, 1984; Jarvis, 1984; Solecka et al., 2008). So far, the regulatory mechanism of PMEI genes in plant cold resistance under low-temperature stress, as a regulator of pectin decomposition, remains unknown.

Watermelon, an important agricultural economic crop belonging to the Cucurbitaceae family, is an annual horticultural crop. Pectin, the main component of the cell wall, is closely related to the thickness of the rind and the tolerance of watermelon to stress (Zhu et al., 2017; Garcia-Lozano et al., 2020). In our previous study, we found that some PMEI genes were downregulated under low-temperature induction. Therefore, the objectives of the present study were to conduct a genome-wide characterization of the PMEI gene family in the watermelon genome and reveal their expression profiling in response to stress. The results of this study not only provide target genes for the study of the PMEI family but also lay a foundation for the investigation of the molecular regulation of pectin methylation in watermelon.

## 2 Materials and methods

### 2.1 Plant materials and treatments

The watermelon seeds (cv. Nongkeda No. 5) were provided by Cucurbit Germplasm Innovation and the Genetic Improvement Laboratory of the College of Horticulture, Northwest A&F University. All seeds were cultured in a greenhouse until three true leaves were available for treatments under the following conditions: 26 ± 2°C, 14 h light, 10 h dark (day/night) photoperiod, photosynthetic photon flux density (PPFD) of 600 µmol m ^−2^ s ^−1^, and a relative humidity of 70–90%. To reveal the potential responses of stress, the watermelon was treated with cold at 4°C for 48 h and unwatered for 8 days. The control group underwent routine management procedures.

Leaf samples were collected at 6 h, 12 h, 24 h, and 48 h after low-temperature treatment and 2 d, 4 d, 6 d, and 8 d after drought conditions. The plant samples at 0 h and 0 days were considered as controls. The leaf, root, stem, tendril, female flower, and male flower samples were collected for tissue-specific expression analysis. These tissue samples were obtained from watermelon plants that had been cultivated and managed under normal conditions. There were three biological replicates for all the collected tissue samples, which were immediately frozen in liquid nitrogen and then stored in a −80°C freezer for further use.

### 2.2 Identification and sequence analysis of *ClPMEI* family members

The newly released watermelon genome was used for the genome-wide identification of PMEI genes and is available at the following website: http://cucurbitgenomics.org/v2. The hidden Markov model (HMM) built based on the PMEI domain (PF04043) against the Pfam database (http://pfam.xfam.org/,version33.1) was used for searching the watermelon protein database by HMMER3.1 (E-value=0.01). The putative *ClPMEI* genes were further checked for the identified candidate homologs using the SMART (http://smart.embl-heidelberg.de) and Pfam (http://pfam.xfam.org) databases. The isoelectric point (pI) and molecular weight (MW) of each ClPMEI protein were predicted using the ExPASy Proteomics Server (https://www.expasy.org). Additionally, the subcellular localizations of these ClPMEI proteins were predicted using the Euk-mPLoc 2.0 server (http://www.csbio.sjtu.edu.cn/bioinf/euk-multi-2/). All the related obtained information of these *ClPMEI* genes are listed in **Supplementary Table S1**.

### 2.3 Chromosome distribution and phylogenetic analysis

According to the physical locations of each gene on the watermelon genome database, these *ClPMEI* genes were mapped onto chromosomes using TBtools software (https://github.com/CJ-Chen/TBtools/%20releases). Based on the previously reported literature, we collected the PMEI proteins of *A. thaliana* from the National Center for Biotechnology Information database (https://www.ncbi.nlm.nih.gov/) (Li Z. et al., 2022). Multiple sequence alignments of all these obtained proteins were performed using CLUSTALW. The phylogenetic tree was constructed using MEGA 11, employing the neighbor-joining (NJ) method with the following specific parameters: the P-distance model, partial deletion with a 50% deletion threshold, and a 1,000-iterations bootstrap test for statistical support.

### 2.4 Gene structure and motif analysis

The 2.0-kb upstream sequences from the ATG transcription start codon of each *ClPMEI* gene were obtained for promoter analysis. The potential cis-regulatory elements were analyzed using the PlantCARE online tool (http://bioinformatics.psb.ugent.be/webtools/plantcare/html). Conserved motif structures were predicted using Multiple EM for Motif Elicitation (MEME, http://meme-suite.org/), in which the parameter of a motif width larger than 10 and less than 50 was used. The exon-intron structures of *ClPMEI* genes were analyzed and plotted with GSDS2.0 software [Gene Structure Display Server 2.0 (gao-lab.org)].

### 2.5 RNA isolation and gene expression analysis

Total RNA was isolated from samples using a Plant RNA Kit (GENENODE, Beijing, China) following the manufacturer’s instructions. Subsequently, the first-strand cDNA synthesis was performed using a FastKing RT Kit with gDNase (TIANGEN). The gene-specific primers used for quantitative real-time PCR (qPCR) analysis were designed with Primer 6 Input and are shown in **Supplementary Table S4**. Quantitative PCR was conducted using the 2 × SYBR Green Premix in a volume of 20 µl (ChamQ SYBR Qpcr Master Mix, Nanjing, China). The procedures were as follows: 95°C for 3 min; 95°C for 15 s, 60°C for 30 s, with 40 cycles in a IQ5 Multicolor Real-Time PCR Detection System (Bio-Rad). Relative quantification was calculated according to the 2^-ΔΔCT^ method described by Livak and Schmittgen (2001). Actin was used as an internal control. Each PCR assay was run with three independent biological and technological replicates. The expression levels of each gene among different tissues were displayed with a heatmap using TBtools software (https://github.com/CJ-Chen/TBtools). The significance tests between the control and the treatments were carried out using a t-test analysis. One-way analysis of variance (ANOVA) was performed using SPSS 26, and multiple comparisons were performed using Duncan’s test to analyze differences at the 0.05 significance level.

### 2.6 Verification of subcellular localization

The full-length coding sequences of ClPMEI genes without stop codons were cloned into the vector pGreen 0229. The vector pGreen 0229 contained the green fluorescent protein (GFP) to generate GFP-ClPMEI fusion proteins and pGreen35s::GFP was used as a positive control. The primer sequences for cloning and vector construction are shown in **Supplementary Table S5**. The One Step Seamless Cloning Kit (DT100-10, DiNing, Beijing, China) was used to fuse the ClPMEI gene sequences to the linearized plasmid that was obtained through the double enzyme digestion with Xhol and EcoRV. The recombinant vectors were checked by Sanger sequencing and transformed into *Agrobacterium tumefaciens* strain GV3101 (Psoup-19) competent cells. The *Agrobacterium* cells were infiltrated into the abaxial side leaves of 4- to 6 week-old *Nicotiana benthamiana*. GFP fluorescence in the transformed leaves after 48 h co-infiltration were visualized using a laser scanning confocal microscope (TCS SP8 SR, Leica, Germany).

## 3 Results

### 3.1 The identification and characterization of ClPMEI family members

To identify the PMEI family genes of watermelon, a local search was conducted in the watermelon genome using HMMER3.0 based on the PMEI domain (PF04043). A total of 60 ClPMEI gene family members were identified from the watermelon genome, which were designated as *ClPMEI1*-*ClPMEI60* based on their chromosomal locations. Data including the protein sequence length, molecular weight, theoretical pI, grand average of hydrophobicity (GRAVY), and subcellular localization prediction are shown in **Table 1**. These ClPMEI genes encoded predicted peptides ranging from 116 to 604 amino acids and the molecular weights (Mw) ranged from 65.95 (*ClPMEI6*) to 12.48 (*ClPMEI38*) kDa. The isoelectric point (pI) values of ClPMEI gene family members ranged from 10.11 (*ClPMEI26*) to 4.05 (*ClPMEI10*). Most *ClPMEIs* were predicted to be hydrophilic, with the grand average of hydropathicity (GRAVY) values below zero. The predicted localization of most ClPMEI homologs include the cell wall, cell membrane, and cytoplasm. The protein sequence details of all family members are shown in **Supplementary Table S1**.

**Table 1:** ClPMEI genes in watermelon.

Gene name	Gene ID	Protein length (aa)	Molecular weight (kDa)	pI	GRAVY	The instability index (II)	Subcellular location(s)
*ClPMEI1*	*Cla97C01G000480.1*	204	22.0375	9.85	0.037	49.16	Cell wall
*ClPMEI2*	*Cla97C01G003880.1*	210	22.953	6.28	-0.135	39	Cell membrane
*ClPMEI3*	*Cla97C01G003890.1*	192	20.78775	4.92	0.027	38.13	Cell membrane. Cytoplasm
*ClPMEI4*	*Cla97C01G004980.1*	245	26.82194	9.2	0.127	99.59	Cell wall
*ClPMEI5*	*Cla97C01G006690.1*	199	21.55763	9.41	-0.105	38.55	Cell wall
*ClPMEI6*	*Cla97C01G006700.1*	604	65.95291	9.32	-0.157	41.06	Cell membrane
*ClPMEI7*	*Cla97C01G007540.1*	197	21.44064	9.75	-0.072	37.34	Cell wall
*ClPMEI8*	*Cla97C01G007550.1*	604	65.6153	8.6	-0.12	41.37	Cell wall
*ClPMEI9*	*Cla97C01G007560.1*	218	23.72708	9.32	-0.034	43.62	Cell wall
*ClPMEI10*	*Cla97C01G021790.1*	197	21.36603	4.05	-0.073	43.42	Cell wall
*ClPMEI11*	*Cla97C02G026840.1*	195	21.41265	9.51	-0.147	38	Cell membrane
*ClPMEI12*	*Cla97C02G026860.1*	186	21.14937	9.1	-0.32	53.01	Cell wall
*ClPMEI13*	*Cla97C02G027500.1*	190	21.35269	6.81	-0.143	38.03	Cell wall
*ClPMEI14*	*Cla97C02G040540.1*	588	64.30679	8.7	-0.349	25.93	Cell membrane. Extracell.
*ClPMEI15*	*Cla97C02G040550.1*	570	63.10493	6.87	-0.308	32.07	Cell membrane. Extracell.
*ClPMEI16*	*Cla97C03G054320.1*	150	16.08424	4.4	0.024	24.16	Cell membrane
*ClPMEI17*	*Cla97C03G057500.1*	594	65.73703	8.51	-0.366	37.37	Cell wall
*ClPMEI18*	*Cla97C03G065030.1*	186	20.6987	8.25	-0.156	43.31	Cell membrane. Cytoplasm.
*ClPMEI19*	*Cla97C03G067530.1*	241	26.28905	5.58	-0.188	59.72	Cell wall
*ClPMEI20*	*Cla97C03G067540.1*	524	58.45619	8.83	-0.301	30.31	Cell wall
*ClPMEI21*	*Cla97C03G067560.1*	586	64.76762	9.27	-0.273	28.74	Cell membrane
*ClPMEI22*	*Cla97C04G079400.1*	567	62.18915	8.41	-0.241	30.27	Cell membrane
*ClPMEI23*	*Cla97C05G082930.1*	566	62.23804	9.2	-0.25	33.14	Cell wall
*ClPMEI24*	*Cla97C05G082940.1*	568	62.66846	6.07	-0.28	29.47	Cell wall
*ClPMEI25*	*Cla97C05G083460.1*	576	63.4819	7.99	-0.255	39.66	Cell membrane. Extracell.
*ClPMEI26*	*Cla97C05G087810.1*	533	59.43461	10.11	-0.123	38.85	Cell wall
*ClPMEI27*	*Cla97C05G089160.1*	177	19.63249	9.07	-0.236	47.52	Cell wall
*ClPMEI28*	*Cla97C05G089170.1*	568	62.49173	7.93	-0.254	27.91	Cell membrane. Cytoplasm.
*ClPMEI29*	*Cla97C05G089190.1*	567	62.30218	9.24	-0.155	30.13	Cell membrane
*ClPMEI30*	*Cla97C05G091180.1*	541	61.23958	8.72	-0.288	42.16	Cell wall
*ClPMEI31*	*Cla97C05G103840.1*	568	62.25397	9.13	-0.244	32.41	Cell wall
*ClPMEI32*	*Cla97C05G107730.1*	567	61.8546	9.02	-0.197	30.6	Cell membrane. Cytoplasm.
*ClPMEI33*	*Cla97C05G107740.1*	509	55.04853	9.16	-0.143	33.78	Cell membrane. Extracell.
*ClPMEI34*	*Cla97C05G107750.1*	555	60.82718	9.1	-0.052	26.31	Cell membrane. Extracell.
*ClPMEI35*	*Cla97C06G110100.1*	188	21.43187	8.84	-0.102	48.92	Cell membrane. Cytoplasm.
*ClPMEI36*	*Cla97C06G110110.1*	168	17.61098	4.71	0.137	23.45	Cell membrane. Extracell.
*ClPMEI37*	*Cla97C06G110120.1*	515	56.70309	6.4	-0.188	33.84	Cell membrane. Extracell.
*ClPMEI38*	*Cla97C06G114010.1*	116	12.47618	4.31	-0.073	38.95	Cell membrane. Extracell.
*ClPMEI39*	*Cla97C06G114020.1*	542	59.3921	6.23	-0.121	38.49	Cell membrane
*ClPMEI40*	*Cla97C06G114030.1*	182	19.54027	6.28	0.014	30.04	Cell membrane. Extracell.
*ClPMEI41*	*Cla97C06G114040.1*	279	29.33297	4.37	-0.019	70.5	Cell membrane
*ClPMEI42*	*Cla97C06G114450.1*	568	62.55273	7.85	-0.199	33.47	Cell wall
*ClPMEI43*	*Cla97C06G125610.1*	363	40.65088	8.83	-0.099	39.74	Cell membrane
*ClPMEI44*	*Cla97C07G141120.1*	151	16.49088	6.71	-0.06	66.09	Cell wall
*ClPMEI45*	*Cla97C07G141130.1*	449	49.6233	9.31	-0.225	25.42	Cell wall
*ClPMEI46*	*Cla97C08G161540.1*	589	64.25855	7.93	-0.226	41.41	Cell membrane. Extracell.
*ClPMEI47*	*Cla97C09G163490.1*	555	60.91557	9.07	-0.249	32.86	Cell wall
*ClPMEI48*	*Cla97C09G168010.1*	187	19.90868	4.5	0.202	18.09	Cell wall
*ClPMEI49*	*Cla97C09G168020.1*	557	61.02362	8.81	-0.198	30.38	Cell wall
*ClPMEI50*	*Cla97C09G168370.1*	160	17.22663	6.36	-0.062	23.96	Cell membrane
*ClPMEI51*	*Cla97C09G171500.1*	160	17.05937	5.24	-0.036	28.19	Cell wall
*ClPMEI52*	*Cla97C09G171650.1*	163	17.41313	7.6	0.256	38.01	Cell membrane. Extracell.
*ClPMEI53*	*Cla97C09G175280.1*	508	56.19687	6.37	-0.111	32.26	Cell membrane. Extracell.
*ClPMEI54*	*Cla97C10G184580.1*	183	20.24707	4.99	-0.131	45.04	Cell wall
*ClPMEI55*	*Cla97C10G187580.1*	555	60.52662	6.18	0.021	32.84	Cell membrane. Extracell.
*ClPMEI56*	*Cla97C10G190170.1*	507	56.17161	9.03	-0.196	38.44	Cell wall
*ClPMEI57*	*Cla97C10G193990.1*	151	16.33741	5.18	-0.372	52.26	Cell membrane. Extracell.
*ClPMEI58*	*Cla97C10G194330.1*	481	53.02236	9.11	-0.19	33.15	Cell membrane. Nucleus.
*ClPMEI59*	*Cla97C11G206510.1*	187	20.26245	9.53	-0.2	31.53	Cell wall
*ClPMEI60*	*Cla97C11G220530.1*	201	22.65091	4.76	0.067	29.82	Cell wall

**Figure 1:**
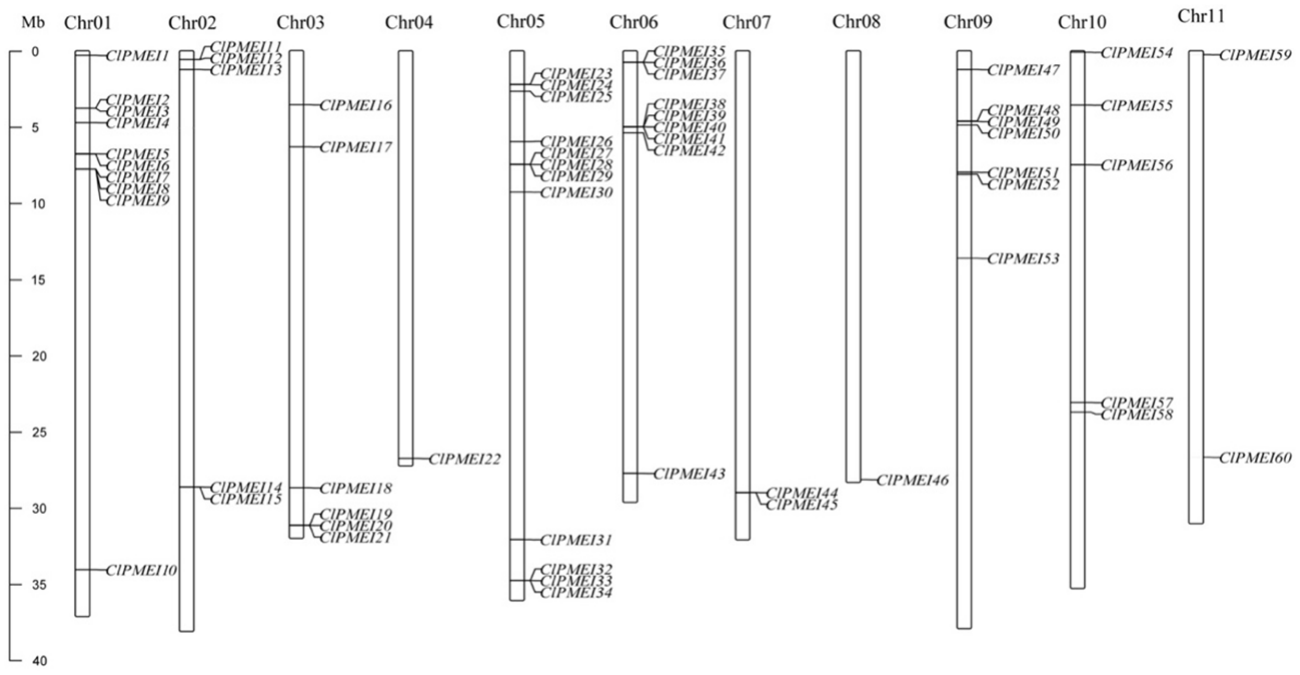
Distribution of ClPMEI genes on watermelon chromosomes. The left scale represents the length of the watermelon chromosomes [megabase (Mb) pairs].

We described the distributions of the ClPMEI homologs, using TBtools, and the physical location in the watermelon genome (**Figure 1**). All the ClPMEI gene family members were anchored onto eleven chromosomes, with Chr04 and Chr08 each harboring only one member. The most ClPMEI members were located onto Chr01, Chr05, Chr06, and Chr09.

### 3.2 Phylogenetic, chromosomal localization, and gene duplication analysis of ClPMEI family members

**Figure 2:**
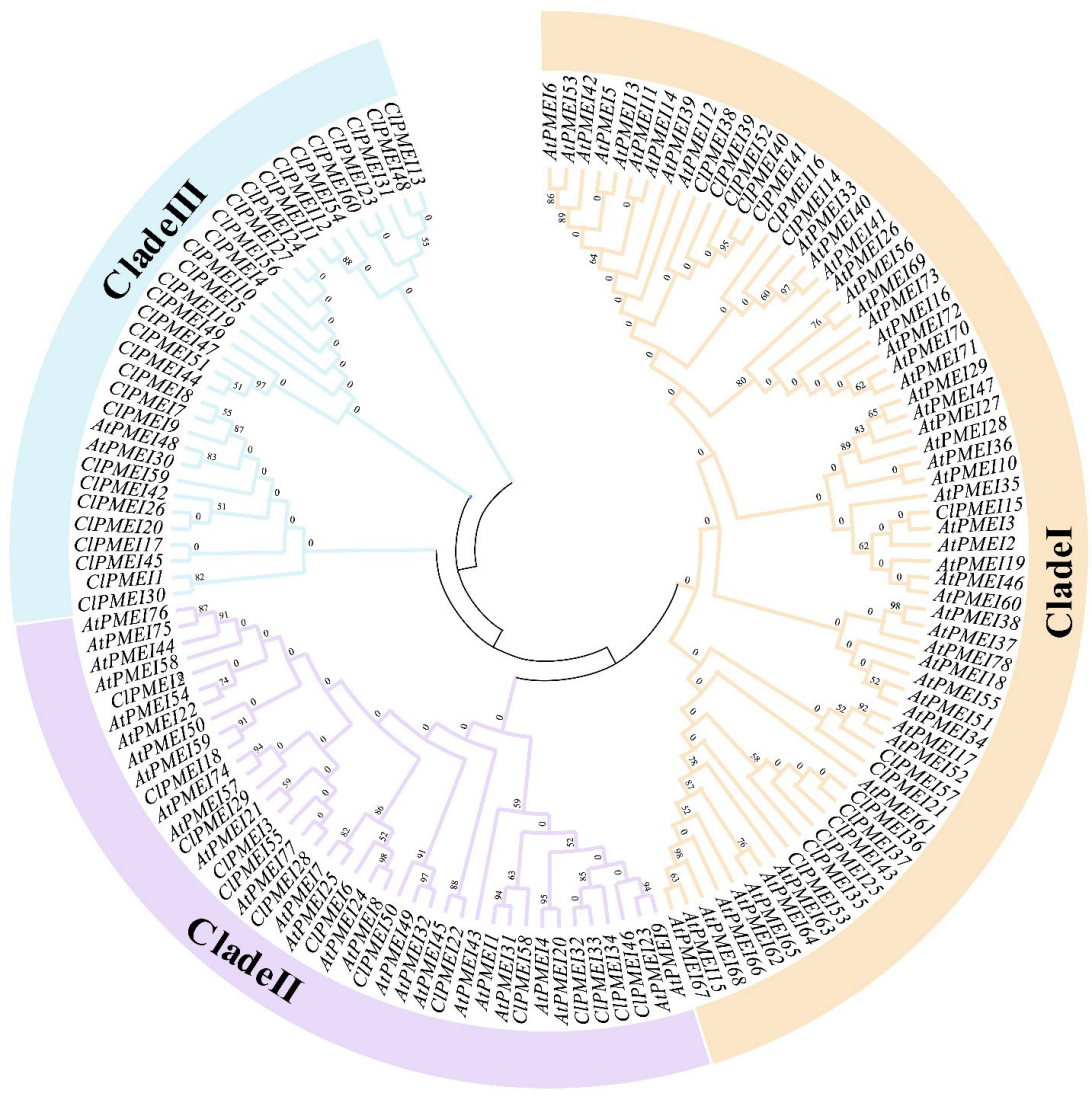
Interspecific phylogenetic tree of PMEI proteins from watermelon and *A. thaliana*. The phylogenetic tree was constructed using the neighbor-joining (NJ) method with a bootstrap test (1,000 iterations) by MEGA X.

To further clarify the evolution of the ClPMEI gene family, a phylogenetic tree was constructed based on the alignment of protein sequences from *A. thaliana* and watermelon using MEGA-11 (**Figure 2**). These ClPMEI proteins were divided into three clades: Class I, Class II, and Class III. Class I included 16 ClPMEI members, Class II included 14 members, and the remaining 30 members belonged to Class III.

**Figure 3:**
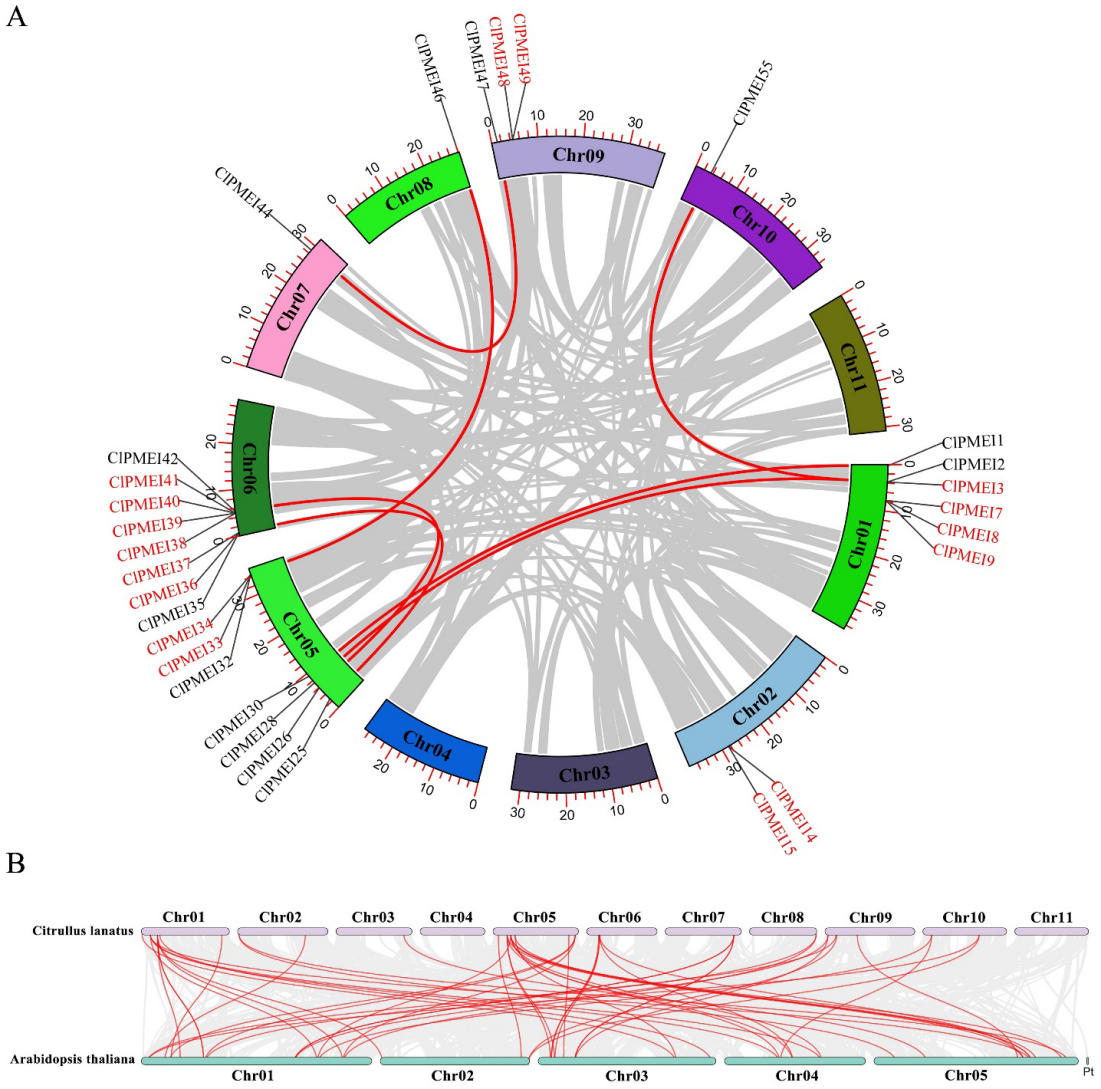
Chromosomal localization, gene duplication, and co-linearity analyses of ClPMEI genes. **(A)** Genome chromosomal localization and duplicated gene pairs of ClPMEI genes in watermelon. The tandemly duplicated genes are indicated in red and the segmentally duplicated genes are indicated in black. **(B)** The syntenic relationship of ClPMEI genes between watermelon and *A. thaliana*. The red curved lines represent orthologous gene pairs between watermelon and *A. thaliana*.

Gene duplication analysis suggested that 16 members were tandemly duplicated genes and 13 were segmentally duplicated genes (**Figure 3A**). Genomic chromosomal localization analysis showed that there were 29 ClPMEI genes distributed on eight chromosomes except Chr 3, 4, and 11. The number of ClPMEI genes on Chr6 was eight, which was the largest number, followed by Chr 5 (seven). Six ClPMEI genes were clustered on Chr 1. Only one ClPMEI gene each was distributed on Chr 7, 8, and 10. On the top region of Chromosome 5, the number of gene clusters reached 4, while in the bottom region, 3 genes formed clusters. On Chr 1, 6, and 9, all genes were distributed in the top region.

Moreover, a syntenic relationship analysis was conducted to further investigate the homology between *A. thaliana* and watermelon (**Figure 3B**; **Supplementary Table S2**). A total of 20 homologous PMEI genes were found in watermelon and *A. thaliana*, which were clustered on Chr1 and 5 in watermelon. In particular, there was a phenomenon in which ClPMEI genes typically matched several *A. thaliana* PMEI orthologous genes, e.g., *ClPMEI2* was homologous with *AtPMEI21*, *AtPMEI54*, *AtPMEI57*, and *AtPMEI74*, and *ClPMEI28* was homologous with *AtPMEI21*, *AtPMEI57*, *AtPMEI54*, *AtPMEI74*, and *AtPMEI75*.

### 3.3 Conserved motif and gene structure analyses of ClPMEI family members

**Figure 4:**
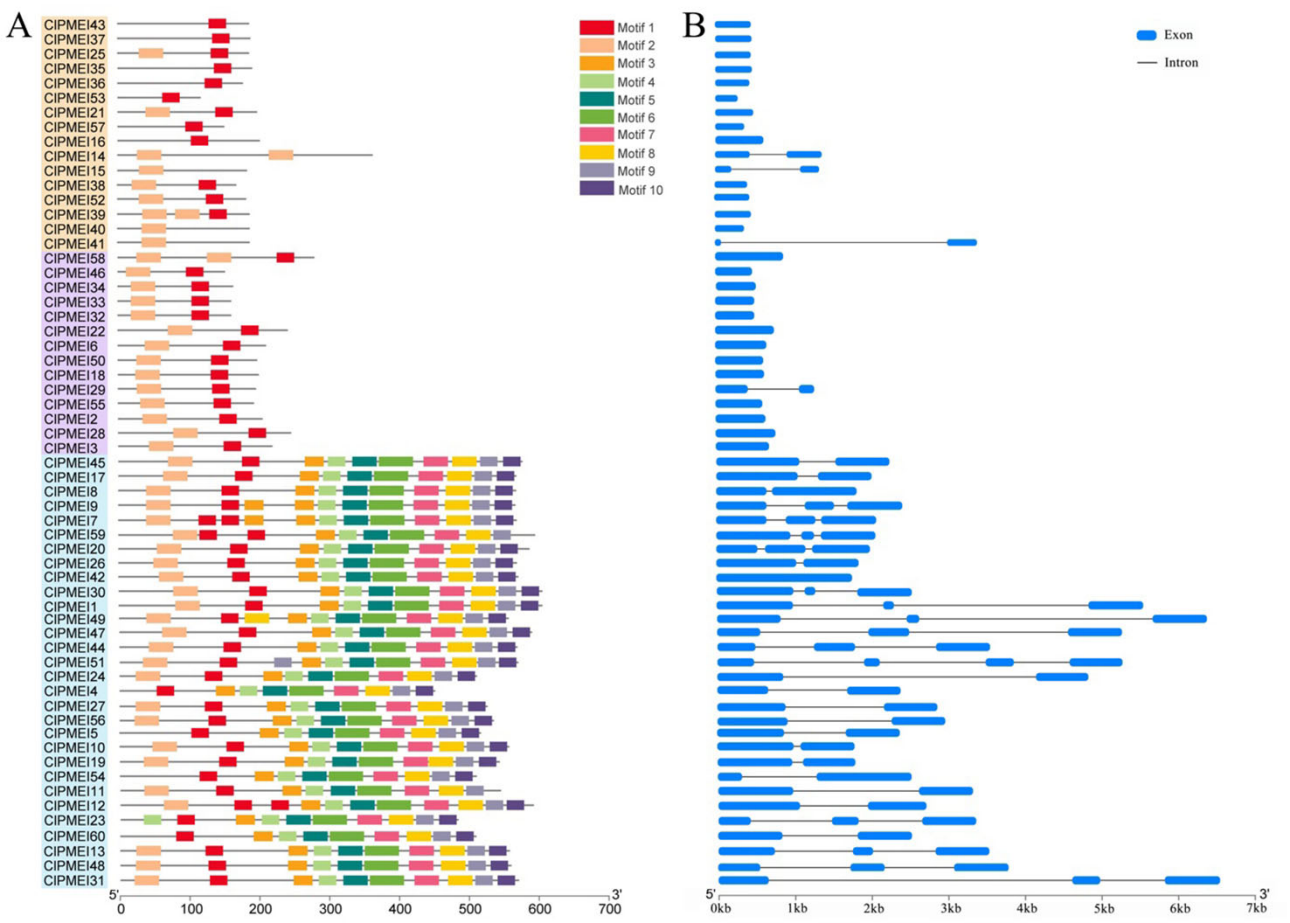
Conserved motif and gene structure analyses of ClPMEI genes in watermelon. **(A)** The distribution of conserved motifs identified from ClPMEI genes. **(B)** The exon-intron structures of ClPMEI genes. Exons and introns are indicated by blue rectangles and lines, respectively.

To predict the potential functions of ClPMEI gene family members, 10 conserved motifs were formed using MEME (**Figure 4A**). Motif 1 and motif 2 correspond to the typical PMEI protein domains and all the information regarding these motifs is listed in **Supplementary Table S3**. Each of the ClPMEI members in Class I contained typical motif 1 or motif 2; either contained two typical motifs. Similarly, all of the members in Class II comprised these two typical motifs. Different from Class I and II, the family members in Class III contained not only the typical motif but also the remaining eight motifs (motifs 3 to 10). Additionally, intron-exon analyses were performed using GSDS2.0 (http://gsds.gao-lab.org/) (**Figure 4B**). Most *ClPMEI* members that belonged to Class I and II only had one exon. The exon numbers of the *ClPMEI* members belonging to Class III ranged from one to four, in which *ClPMEI42* had one exon.

**Figure 5:**
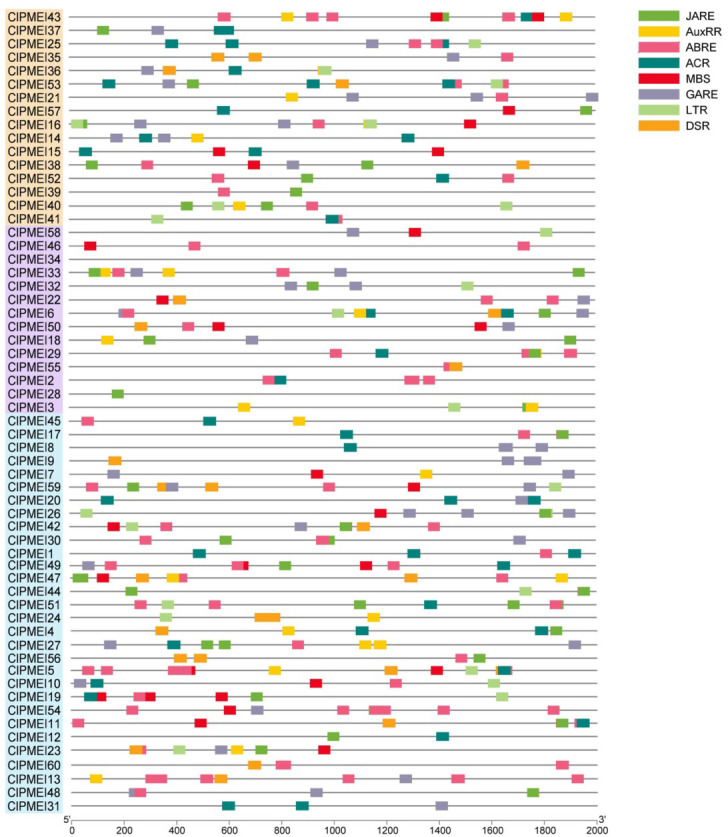
Putative cis-elements in the promoter regions of PMEI genes in watermelon. Different colored rectangles denote different cis-elements with various biological functions.

To reveal the functions and regulatory mechanisms of the ClPMEI gene family, the 2.5 kb genomic sequence upstream of the translation start site was retrieved for each ClPMEI gene and analyzed using the PlantCARE online tool. The cis-elements are listed in **Figure 5**, including development-related, stress response-related, and phytohormone-related cis-elements. According to the results, up to 40 family members have salicylic acid responsiveness elements (ACR), 34 have MeJA-responsiveness elements (JARE), and 32 have gibberellin-responsiveness elements (GARE) in the promoter sequences. Moreover, the low-temperature responsiveness cis-element (LTR) and defense and stress responsiveness cis-element (DSR), which are related to defense and stress, were also presented in the promoters of ClPMEIs.

### 3.4 The expression patterns of ClPMEI genes under abiotic stress

To reveal the potential regulatory models of ClPMEI genes, the expression patterns of family members were checked under abiotic stress. A total of 19 ClPMEI genes were randomly selected from three classes for quantitative analysis. The results showed that the expression levels of ClPMEI genes under low-temperature and drought treatments exhibited different patterns.

**Figure 6:**
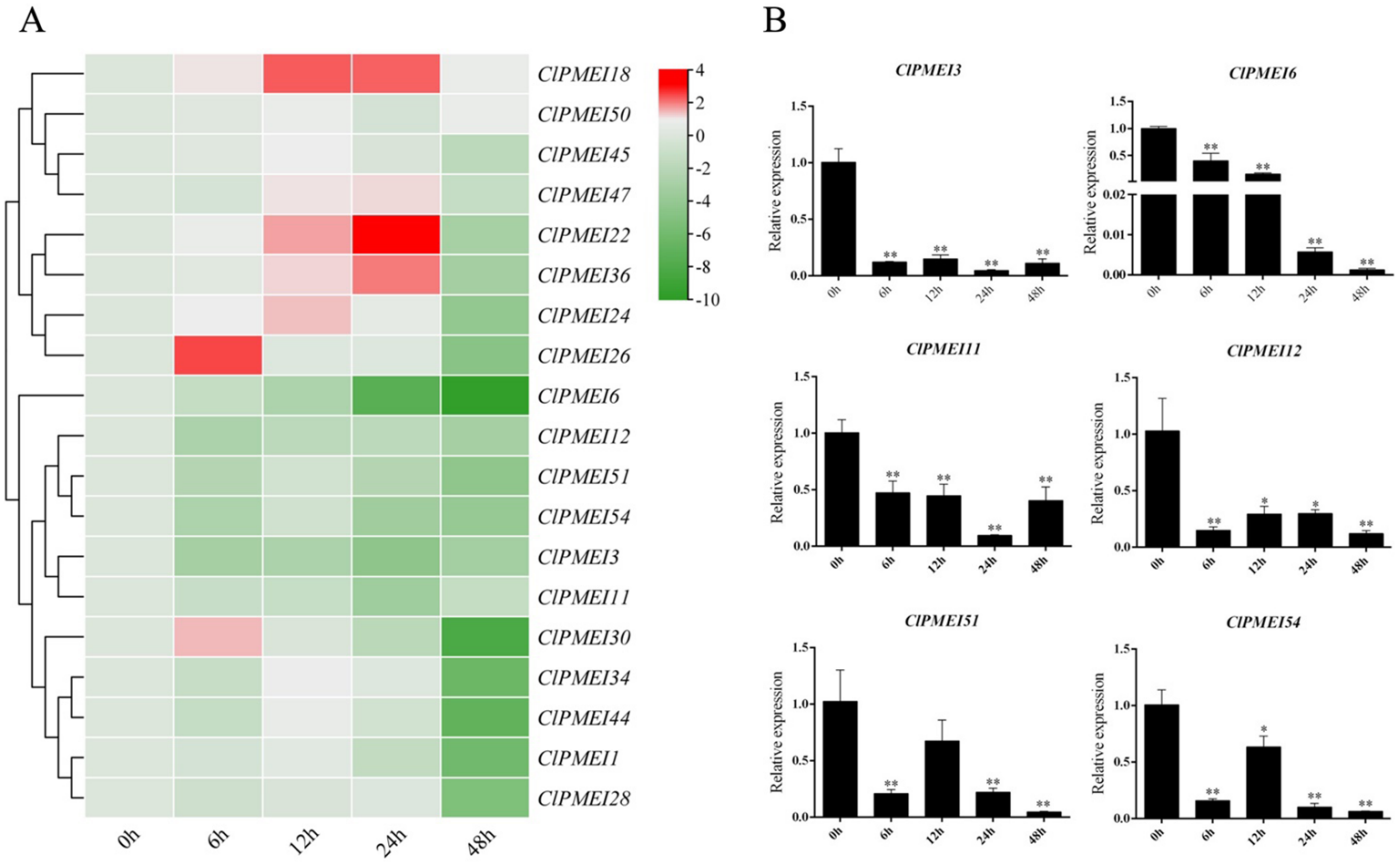
Expression analysis of PMEI genes in watermelon under low-temperature treatments. **(A)** Gene expression heatmap of PMEI genes. The bars of the heatmap represent expression values after log2 transformation and qRT-PCR expression values normalization. The color scale from green to red represents the lower to higher relative expression levels. **(B)** Relative expressions of ClPMEI3, 6, 11, 12, 51 and 54 under low-temperature. Column cluster analysis shows different groups based on the expression. Student’s t-test was used to determine significant differences at the same period between control group and treatment group. Significance level: * P<0.05. ** P<0.01.

The results showed that, under the low-temperature treatment, the relative expression levels of most of the tested family members showed downregulated trends, with the exception of *ClPMEI18*, the expression level of which showed obvious increases compared with the controls at all sample points, with the most significant increase observed 12 and 24 h after treatment (**Figure 6A**). The expression levels of *ClPMEI22*, *ClPMEI24*, *ClPMEI36*, and *ClPMEI47* were upregulated in samples that were subjected to low temperatures for 12 and 24 h. Compared with the control groups, *ClPMEI26* and *ClPMEI30* showed relatively higher expression levels 6 h after low-temperature treatment. The expression levels of *ClPMEI3*, *ClPMEI6*, *ClPMEI1*, *ClPMEI12*, *ClPMEI51*, and *ClPMEI54* showed downregulated trends compared with the control groups in the low-temperature conditions (**Figure 6B**). *ClPMEI3*, *ClPMEI6*, and *ClPMEI11* were significantly decreased compared with the control group at all sampling times. Notably, for ClPMEI family members, an evident decrease was observed at 48 h under the low-temperature treatment; some members were significantly decreased at this point.

**Figure 7:**
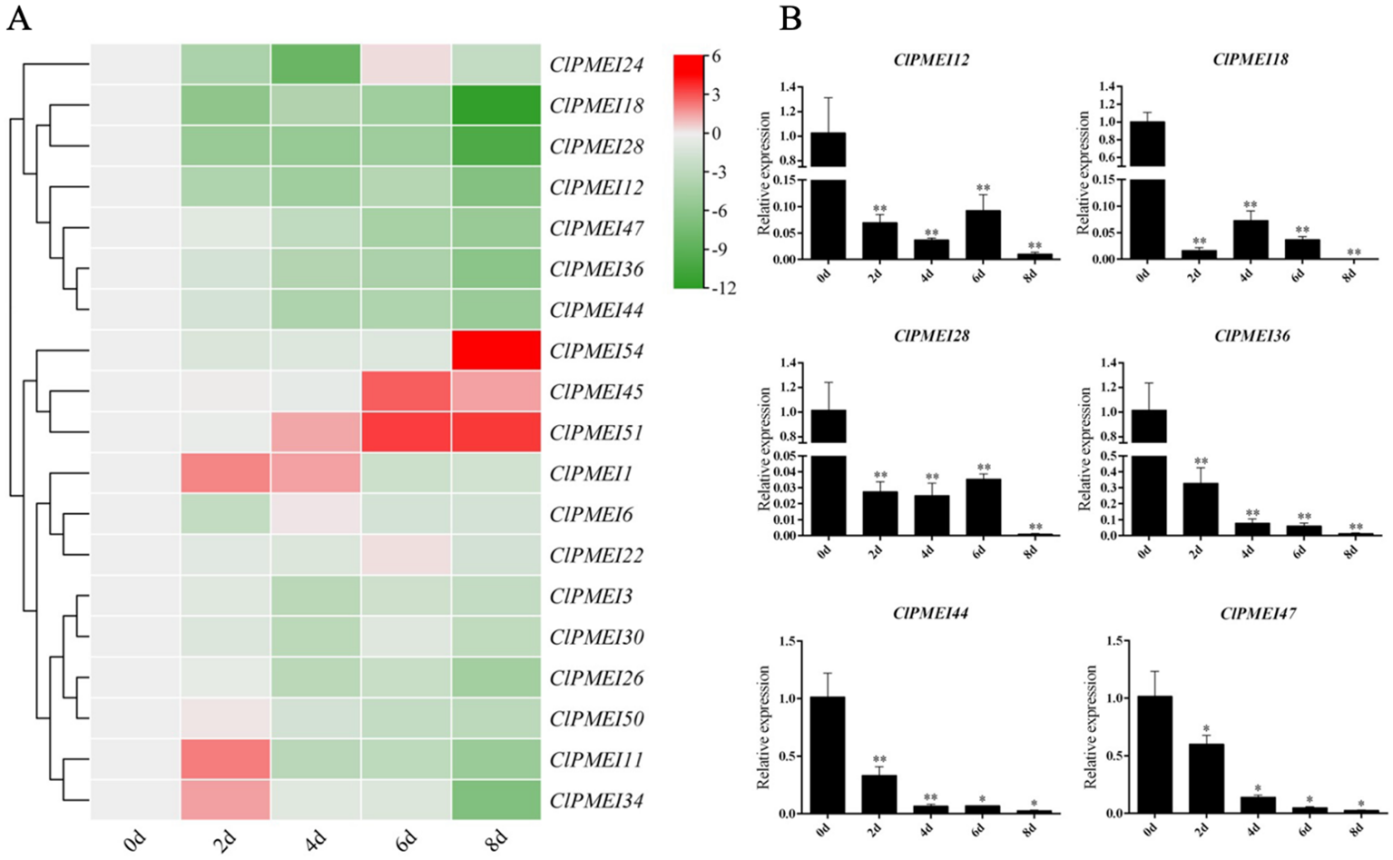
Expression analysis of PMEI genes in watermelon under drought treatments. **(A)** Gene expression heatmap of PMEI genes. The bars of the heatmap represent expression values after log2 transformation and qRT-PCR expression values normalization. The color scale from green to red represents the lower to higher relative expression levels. **(B)** Relative expressions of ClPMEI12, 18, 28, 36, 44 and 47 under drought treatments. Column cluster analysis shows different groups based on the expression. Student’s t-test was used to determine significant differences at the same period between control group and treatment group. Significance level: * P<0.05. ** P<0.01.

For the drought conditions, the expression levels of most ClPMEI gene family members were downregulated compared with the controls (**Figure 7A**). The expression of *ClPMEI11*, *ClPMEI34*, and *ClPMEI50* showed an increase compared with their controls 2 days after treatment. The upregulated expression of *ClPMEI1* appeared 2 days after treatment and continued until the fourth day, after which it was downregulated. *ClPMEI45* showed a downregulation of expression compared with the controls 2 and 4 days after treatment, followed by a significant upregulation on the sixth and eighth days after treatment. Similarly, after continuous downregulation, the expression of *ClPMEI54* showed significant upregulation 8 days after treatment compared with the control. In addition, *ClPMEI51* showed a gradual upward trend in expression as the treatment time increased from the fourth to the eighth day after treatment. Similar to the results obtained from the low-temperature treatment, most of these family members showed a downregulation under drought treatment. The expression levels of *ClPMEI12*, *ClPMEI18*, *ClPMEI28*, *ClPMEI36*, *ClPMEI44*, and *ClPMEI47* showed a significant downregulation at all sampling times under drought treatment (**Figure 7B**). *ClPMEI12*, *ClPMEI18*, *ClPMEI28*, and *ClPMEI36* were significantly decreased compared with the control group at all sampling times.

### 3.5 Expression patterns of ClPMEI genes in different watermelon tissues and the subcellular localization of ClPMEI proteins

**Figure 8:**
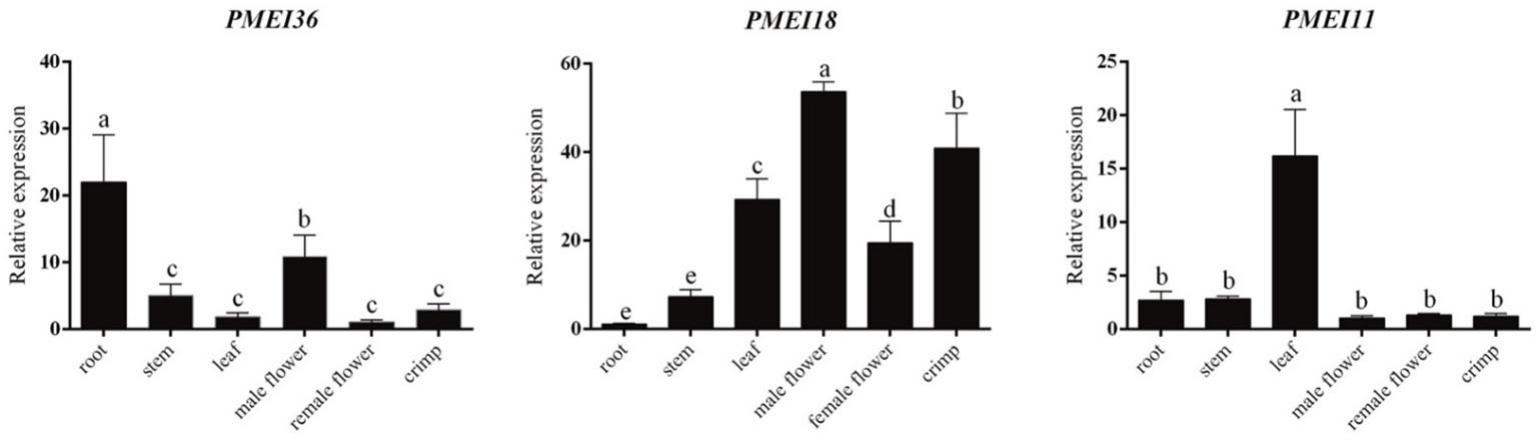
Expression analysis of PMEI genes in different watermelon tissues. Different letters represent statistically significant differences by SPSS (ANOVA with Tukey *post-hoc* analysis, 5% level).

To primarily investigate the functions of ClPMEI genes, three family members were randomly selected from three clades to analyze the expression patterns among six different watermelon tissues. The tissues included root, stem, leaf, male flower, female flower, and crimp. As shown in **Figure 8**, *ClPMEI11* was highly expressed in leaves but exhibited relatively low expression in other tissues. The expression of *ClPMEI18* in male flowers and crimp was higher than other tissues; however, the gene was rarely expressed in root. *ClPMEI36* was highly expressed in root and male flowers.

**Figure 9:**
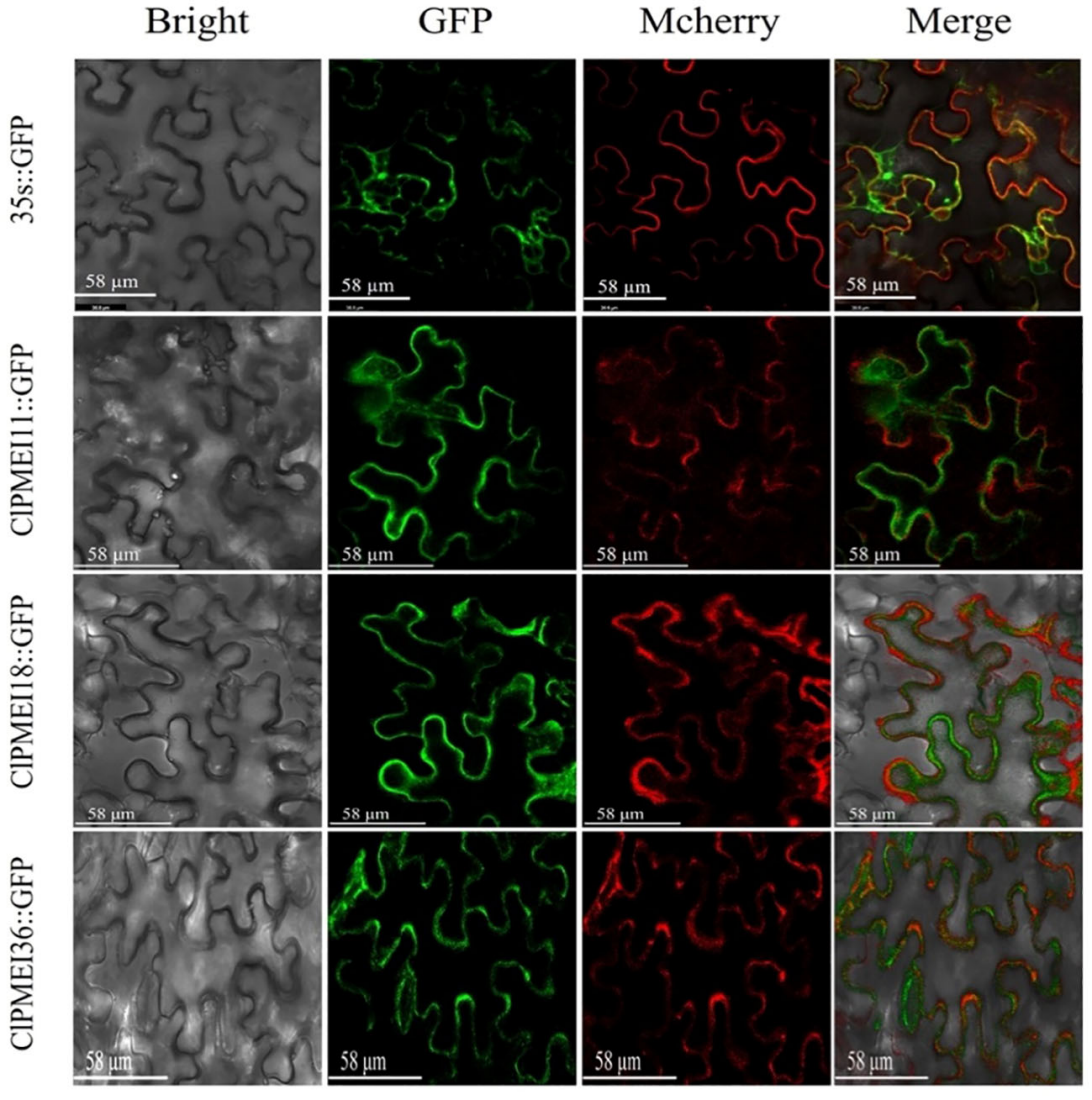
Subcellular localization of GFP-fused ClPMEI11, ClPMEI18, and ClPMEI36 in tobacco leaves observed by fluorescence microscopy. Leaves expressing 35S::GFP alone were used as positive controls. Scale bars: 58 μm. Bright-field, green fluorescence, mcherry (plasma membrane marker), and merged images are shown from left to right.

To verify the results of the predicted subcellular localization, these three family members were also selected for verification. As shown in **Figure 9**, the subcellular localizations of these proteins were verified with the transient expression of the GFP fusion proteins ClPMEI11-GFP, ClPMEI18-GFP, and ClPMEI36-GFP in tobacco leaf cells. Consistent with the predicted results, the fluorescence signal of GFP could be detected in the cell membrane of tobacco cells.

## 4 Discussion

As an important component of the cell wall, pectin plays an important role in plant growth, communication response, and so on. HG, as a major subclass of pectin, affects the biological functions of the cell wall through its synthesis and metabolism. PMEI is a large protein superfamily that regulates HG methylation and pectin hardness by participating in the post-transcriptional regulation of PME. The interaction between PMEI and PME regulates the formation of the primary cell wall and thus fruit hardness. The PMEI family has been identified in several species, including not only *A. thaliana*, *O. sativa* and *B. oleracea* but also *Citrus sinensis* Osbeck (45 CsPMEIs), *P. bretschneideri* (55 PbrPMEIs), tea plants (51 CsPMEIs), and others (Li B. et al., 2022; Zhu et al., 2021; Li Z. et al., 2022). In this study, 60 ClPMEI gene family members were identified in the cucurbitaceae crop database using a hidden Markov model.

The analysis of the characteristics of ClPMEI family members predicted that most of them are located on the cell membrane. There are also reports of the subcellular localization of PMEI family members in other plants. In citrus, CsPMEI19 was located in the cytoplasm and CsPMEI32 was localized in the plasma membrane (Li et al., 2022). In *A. thaliana*, AtPMEI12 is also reported to be located in the cell wall (Röckel et al., 2008; Lionetti et al., 2017). The different subcellular localizations of PMEI family members is closely related to their functions in plant growth activities. The subcellular localization of ClPMEI was verified, which showed that the three genes were located on the cell membrane, consistent with our prediction. However, this study only randomly selected one member from each of the three subfamilies for verification, which cannot demonstrate the accuracy of the prediction results.

In some plants, researchers have found that hormones are involved in regulating the expression of PMEI genes. For example, PMEI in wheat is induced by salicylic acid and jasmonic acid (Hong et al., 2010). The transcription of PMEI1 in pepper was activated by abscisic acid (An et al., 2008). Similarly, in this study, there were 38 promoters with abscisic acid and auxin-related cis-elements among 60 ClPMEI family members. The promoter sequences of ClPMEIs were analyzed and showed that there were several plant hormone response elements in the promoter sequences of ClPMEIs. These response elements reflected that the PMEI gene family in watermelon may have a similar situation, and its expression is regulated and activated by plant hormones. In addition, we also found stress-related response elements in the promoter sequences of PMEI genes in watermelon.

*PMEI* genes have been reported to be involved in plant responses to environmental changes. Plants respond to abiotic stresses, such as water deficit, salt stress, and extreme temperatures, by altering their cell wall structure. Overexpression of *CaPMEI1* in *A. thaliana* showed increased drought tolerance (An et al., 2008). RT-PCR analysis of PMEI genes in wheat showed that they also respond to water stress, and this response is mediated by polyethylene glycol (Hong et al., 2010). In this study, we analyzed the expression pattern of the ClPMEI gene family in watermelon under drought conditions. Although the expression trends of genes in different family members vary under drought conditions, we infer from this result that the ClPMEI genes respond to drought stress. The expression levels of *ClPMEI45*, *51*, and *54* were upregulated after drought treatment. In particular, *ClPMEI51* and *54* showed a significant upregulation in expression levels during the late stage of drought treatment, suggesting that they may play a positive regulatory role in the drought stress response in watermelon (**Supplementary Figure S1**).

Similarly, specific transcriptional regulation of PMEI genes has also been observed under low-temperature conditions. Under cold conditions, this negative regulatory mode of PMEIs might lead to an increase in pectin levels in plant cell walls, further reducing cell wall porosity and increasing cell adhesion to balance the direct relationship between freezing resistance and growth (Solecka et al., 2008). Overexpression of the homologous gene *CbPMEI1* in the highly cold-tolerant alpine plant *Chorispora bungeana* resulted in decreased freezing resistance in transgenic plants (Baldwin et al., 2014). In this study, the expression levels of *ClPMEI3*, *ClPMEI6*, *ClPMEI11*, *ClPMEI12*, *ClPMEI51*, and *ClPMEI54* showed a significant downregulation compared with the controls under low-temperature induction. Combined with similar experimental results, we can speculate that members of the PMEI gene family in watermelon play a negative regulatory role in low-temperature stress. Intriguingly, the same ClPMEI genes exhibit different expression patterns under different treatments in watermelon. For example, *ClPMEI18* and *ClPMEI50* exhibit opposite expression patterns under low-temperature and drought treatment. The expression level is upregulated under low-temperature induction and significantly suppressed under drought induction. This phenomenon suggested that different ClPMEI genes exhibit specific functions under different abiotic stresses. In addition, based on the analysis of the quantitative results, it was found that both *ClPMEI51* and *ClPMEI54* exhibited positive regulatory effects under low-temperature and drought treatments. These two genes can be used as research objects to study the relationship between watermelon ClPMEI genes and abiotic stress resistance. Despite this, more research is needed to reveal the detailed functions of ClPMEIs in watermelon from the perspective of abiotic stress response.

## 5 Conclusion

In this study, 60 ClPMEI genes were identified in the watermelon genome. These ClPMEI gene family members were systematically analyzed in terms of their gene structure, cis-elements, conserved domains, subcellular localization, phylogenetic relationships, chromosomal locations, and expression profiles in the present study. These 60 gene members are distributed on 11 chromosomes. Through analysis of their cis-elements and gene expression profiles, it was found that ClPMEI genes respond to low-temperature and drought conditions. These results suggest that ClPMEI genes are involved in the pathway of watermelon response to abiotic stress. *ClPMEI51* and *ClPMEI54* exhibited positive regulatory effects under both low-temperature and drought treatments. Overall, these research results show that ClPMEIs potentially play a role in regulating watermelon low-temperature and drought resistance and also laid a foundation for further research on the function of watermelon ClPMEI genes.

## Data Availability

The datasets presented in this study can be found in online repositories. The names of the repository/repositories and accession number(s) can be found in the article/**Supplementary Material**.
