# Supplementary material for: Genome-wide identification and characterization of pectin methylesterase inhibitor gene family members related to abiotic stresses in watermelon
Source: Front Plant Sci. 2024 Sep 17;15:1454046. doi: 10.3389/fpls.2024.1454046 (PMC11442291; doi:10.3389/fpls.2024.1454046)
Supplement: Supplementary file 1 [file DataSheet1.docx]

**Supplementary Figure**


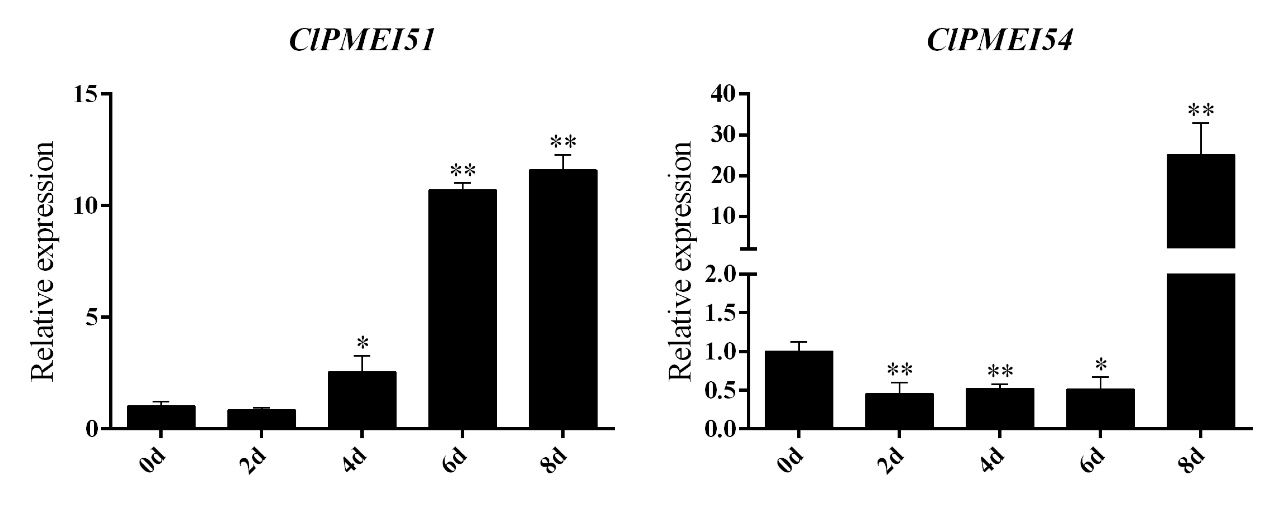


**Fig. S1 Expression analysis of ClPMEI51 and ClPMEI54 in watermelon under drought treatments. Student’s *t*-test was used to determine significant differences at the same period between control group and treatment group. Significance level: * *P*<0.05. ** *P*<0.01.**
